# Devolution and human resources in primary healthcare in rural Mali

**DOI:** 10.1186/1478-4491-9-15

**Published:** 2011-06-08

**Authors:** Elsbet Lodenstein, Dramane Dao

**Affiliations:** 1 Royal Tropical Institute (KIT), Amsterdam, Netherlands; 2 SNV Netherlands Development Organization, Bamako, Mali

## Abstract

Devolution, as other types of decentralization (e.g. deconcentration, delegation, privatization), profoundly changes governance relations in the health system. Devolution is meant to affect performance of the health system by transferring responsibilities and authority to locally elected governments. The key question of this article is: what does devolution mean for human resources for health in Mali?

This article assesses the key advantages and dilemmas associated with devolution such as responsiveness to local needs, downward accountability and health worker retention. Challenges of politics and capacities are also addressed in relation to human resources for health at the local level. Examples are derived from experiences in Mali with a capacity development programme and from case studies of other countries. It is not research findings that are presented, but highlights of key issues at stake aimed at inspiring the debate in Mali and elsewhere.

A first lesson from the discussion suggests that in the context of human resources for health, decentralization of authority and resources is not the main issue. The challenge is to develop or strengthen accountability of those who decide and act, whether they are local politicians, bureaucrats or community representatives. If decentralization policies do not address public accountability, they will not fundamentally change human resource management, quality and equity of staffing. A second lesson is that successful devolution requires innovations in capacity development of all actors involved and in designing effective incentive measures. A final key conclusion is that the topic of devolution policy and its effects on human resources for health, and vice versa, merit more attention. A better understanding may lead to more appropriate policy designs and better preparation for the actors involved in countries that are embarking on decentralization, as is the case in Mali.

## Introduction

Key constraints to health service provision in rural Mali are often linked to resource management, and in particular to the allocation and performance of available human resources. In Mali, the ratio of qualified staff/population is eight times higher in urban areas than in rural health centres, in particular for midwives. In addition to geographical disparities, it is also observed that medical personnel are is not always carrying out their curative role but instead focus on administrative matters [1]. In terms of the availability and quality of staff, rural areas are underserved despite several initiatives to reverse this trend (such as community financing, isolation bonuses and other incentives) and NGO involvement. Currently, the Ministry of Health (MoH) is reviewing its human resources policy with the objective of rationalizing and harmonizing human resources for health.

One of the strategies of the Government of Mali is to decentralize responsibilities for the management of local health centres to local institutions. This is done through two complementary approaches that both aim at increased community involvement, strengthened autonomy and the division of labour (subsidiarity) for increased efficiency. This has been done through (1) delegation of management of health centres to community health associations since 1990 and (2) the devolution of decision-making power to locally elected governments since 2002. Both structures are community-based, elected entities. A community health association (*association de santé communautaire*) is responsible for the daily management and financing of public primary health care clinics. The clinics have a designated catchment area defined by the number of people living within a 5 to 15 km radius from the clinic. "Local governments" (*communes*) in Mali are autonomous entities that consist of locally elected councillors, a mayor and basic administrative staff. They are different from the "local administration" represented by the prefect. Mali has 703 local governments that have an average size of 20 000 inhabitants. The community health association and the local government function as separate structures but representatives from both organizations form the commune health commission that discusses health programmes.

The recent devolution policy implies a transfer of human resources at the primary healthcare level from civil service to local government service, whereby local governments contract local health staff and pay salaries and incentives. Training and performance monitoring remain central tasks of District Health Management Teams (DHMT). In the education sector, this transfer has already taken place; the health sector will embark on this transfer in 2011. Currently, 30% of local health staff is working for local government. This is expected to increase progressively because of the establishment of new health centres and the decentralization of vertical priority programmes, such as HIV/AIDS and tuberculosis control [1]. In the long term, all new staff will work under the local government service.

The rationale for devolution policies in Mali is straightforward but implementation is less so. Decentralized management of human resources is a particularly sensitive topic because it involves diverse political and professional interests. The key questions debated today in Mali relate to governance issues such as authority, accountability and multi-stakeholder competition and interaction.

Due to the relatively recent involvement of local governments in health and a lack of evidence on what works and what does not, discussions on devolution become divisive debates where the arguments of both sides, advocates and opponents, are often equally unfounded.

In the context of this debate, not only in Mali but in many other West African countries, this article will examine the opportunities and pitfalls, real or potential, of devolving human resources for health to the local level. Recent experience in Mali is discussed together with findings from studies done in other countries such as Nigeria, Tanzania, China and Uganda. Although not exhaustive, this article outlines the key issues at stake in Mali and highlights lessons learned from other countries in order to inspire the ongoing debate on devolution. The article is based on a literature review and on insights from a capacity development programme for decentralized management of healthcare in Mali, in particular in the Koulikoro region. The Government of Mali has been implementing this programme since 2004, in collaboration with the Royal Tropical Institute (KIT), the Netherlands Development Organization (SNV) and the Royal Netherlands Embassy in Mali.

## Discussion

### Decentralization and health services, what are we talking about?

As in other countries, the Malian primary healthcare package contains three groups of services: individual-oriented curative services, preventive (outreach) services, and promotional services. Typically local governments are more directly involved in the latter two. In Mali, local governments do not directly manage curative services, but instead delegate management to community health associations. While local governments may not need all the necessary technical capacity themselves, they should be able to participate in health planning and decision-making, budgeting, management processes and performance monitoring. In francophone countries, this role is referred to as "maître d'ouvrage" (contracting authority). Devolution should also be understood in this sense. Regulation and quality assurance remain key tasks of the Ministry of Health's DHMT.

### Devolution and human resources for health: four key issues

Four key opportunities and dilemmas associated with devolution and human resources for health (HRH) are discussed below.

### Responsiveness

Devolution, as well as reforms to improve community participation and client voice, can promote a better fit between services, local conditions and recipient demands [2]. In terms of human resources, a case study in Tanzania found that decentralized recruitment resulted in a more realistic distribution of staff compared to centralized recruitment, where the posting of staff was less responsive to the specific needs of the districts [3].

Local governments in Mali are responsible for local development planning, including social services. They collect statistical data and identify specific needs of the communities through participatory planning methods from village level upward. DHMT has always faced a lack of data for planning and its' staff affirm that accessing basic information through local governments narrows the gap between the identified needs and the allocation of human resources. In addition, DHMT welcomes the initiatives of rural governments to recruit temporary staff according to specific social, economic and ecological conditions. These include the recruitment of additional vaccinators during campaigns, malaria prevention officers in the rainy season and outreach personnel in the agricultural season when people are too busy to travel to health centres.

### Retention of health workers

Decentralization allows local governments to hire staff from within the locality. In Mali, this has resulted in a group of health workers who know their rural living environment and who are less likely to leave for another post. Local governments also offer benefits such as housing or transportation or other incentives in kind. However, these measures appear to be effective only among the lower cadre, not among highly-skilled staff such as medical doctors and nurses. Attracting and retaining doctors and nurses is a major challenge that decentralization policies have not yet been able to resolve. One reason is that local governments and community health associations face fierce competition with central government, which provides civil servant contracts with better security and career perspectives. Consequently, posts in remote areas are mainly used as a bridge to government employment, preferably in more urban areas. This challenge has been noted elsewhere in decentralized systems such as those of the United Republic of Tanzania and China where remote and poorer districts could not compete for qualified staff with central government or with richer local governments, resulting in an uneven quality of service provision [3,4]. Kolehmainen-Aitken [5] further argues that, unless equalization mechanisms are established, competition between poorer and richer local governments may result in inequity in staffing. While the Government of Tanzania opted for a recentralization of recruitment procedures, Mali is currently strengthening decentralized recruitment by harmonizing the status and employee rights of different contracts and regulating competition.

### Downward accountability

Devolution represents the ultimate form of downward accountability by elected local governments to local constituents, and is seen as a way of motivating public providers to improve service delivery [6,7]. So far, evidence on local government accountability is limited or partial, and is mainly based on case studies. In Mali, individual cases have been analysed but a more comprehensive study has yet to be carried out.

After ten years of devolution in Mali, varying degrees of local government accountability are appearing. Some local governments function increasingly as intermediaries between users and service providers. Communities or individual users communicate their needs and complaints with regard to the services offered to their elected councillors, who in turn negotiate with providers or the Ministry of Health to improve performance. Most of these interactions are about the performance of health workers. It is quite common for local authorities to call into question the functioning of a health worker. After investigation of complaints made by the local population, corrective measures are identified, in collaboration with the health facility or through the Ministry of Health.

Representatives of the Ministry of Health in Mali perceive these local monitoring mechanisms as a benefit of devolution. They resolve recurring issues such as staff absences or attitudes, and issues are examined that were difficult for the DMHT to monitor before decentralization. The existence of an elected representative local council can institutionalize the inclusion of client perspectives on quality in a more structured way. Similar perceptions were observed in Uganda, where health staff appreciated the human angle given to supervision, the increased accountability and improved relationships with key community members [8].

However, the decentralization policy itself seems to undermine the emergence of accountability mechanisms in Mali. Currently, responsibilities remain in the hands of several institutions at a time, resulting in parallel lines of accountability. This generates many conflicts that affect health worker motivation. For example, the government pays 1710 health workers on a special fund for Highly Indepted Poor Countries (HIPC), who are contracted by local government and supervised by the Ministry of Health at central level [9]. These workers are accountable to different authorities and function in a vacuum; many of them do not report for work. Similarly, a local government may fire a health worker recruited by the community health association, or the Ministry of Health may remove a medical officer hired by the local government. What remains of downward accountability in this situation? Monitoring and collecting information is one thing, but when it comes to making decisions regarding recruitment, or performance evaluations resulting in rewards or sanctions, the relations get more complicated. In other words, downward accountability cannot be effective without functioning horizontal accountability and upward accountability relations between the different parties involved.

More complete and coherent accountability relations require an alignment of health policies and decentralization policies. In our view, this should be a policy priority in Mali because the institutional confusion could demotivate staff and exacerbate turnover, with serious consequences for health outcomes. The opportunities presented by downward accountability and formal and informal mechanisms for participation should be seized, but the right conditions are needed to make them work. At the operational level, these conditions include the capacities of actors to perform their new tasks under decentralization and their willingness to collaborate and develop effective working relations.

### Capacities

The lack of capacity of local government is a much-debated issue. An argument against decentralization is the lack of financial and human capacities of local institutions. An additional argument in the context of devolution is the lack of political will on the part of elected officials to invest in health and the risk of local elites capturing and redistributing resources through patronage systems. However, in Latin America particularly, some cases show that despite capacity constraints, many interesting innovations in social services have been introduced at the local level, rather than the national level. Nelson [2] argues that investing in health is a potential way of winning political support at the local level, and political parties and citizens are more likely to mobilize around social services at local level than at national level. Key factors that determine priority-setting in service provision are local leaders' values and commitment, the local party system, the social and economic structure, and traditions.

In Mali, personal commitment of local leaders is also a key determinant of the local government's performance in health service delivery. However, without capacity development, incentives and functioning accountability mechanisms, their ability to deliver services is limited. Below, we highlight a few innovations that the Government of Mali has introduced to address this.

#### Social capital

First, in terms of an enabling environment, it should be noted that Mali has a rich experience in community-based development approaches. This is demonstrated, for example, by the early and partly effective implementation of the Bamako Initiative aimed at accelerating primary health care through community mobilization and financing, and the introduction of locally-elected governments from the bottom up. In particular at the operational level, there is a high level of confidence in local initiatives, in health as well as in other sectors. The use and consolidation of "social capital" is essential to operating a social system such as health, and particularly important for decentralized management of health services.

#### Capacity development

Second, the Government of Mali, in particular the Decentralization Unit within the Ministry of Health, with support from the partners mentioned above, has invested strongly in capacity development of actors in the decentralized system. Such programmes were missing in the cases of Tanzania and China, where the actors involved were poorly prepared for decentralization, undermining the efforts (Liu, 2006; Munga 2009). Three key features of the capacity development programme in Mali contribute to its success. First, it has focused not only on the capacities of the "recipients" of responsibilities (local governments) but also on those of the key actors involved in the health system. Decentralization implies a shift of responsibilities and relations between different actors that include policy-makers, regulators, providers, and users, and these need new skills to function and interact. The training programme included modules for the different stakeholders. A second feature of the capacity building programme is that, although it has not yet become national policy, regional health directorates have adopted it and included multi-actor capacity strengthening in their annual programming. And thirdly, the programme builds on existing instruments (planning, supervision, peer review, maternal audits) that are adapted to include participation of local governments and community health associations. This approach builds upon existing capacities, reduces costs and enhances ownership.

#### Financial incentives

Another initiative that the Government of Mali has introduced is financial incentive schemes that aim at motivating service providers and enhancing their capacities to deliver quality health services. The first scheme was the introduction of performance-based financing (PBF) at primary care level in 2010. The main objective is to increase the quantity and quality of health services through performance contracts between local governments and health facilities. Performance-based financing involves a process of joint performance monitoring, planning, benchmarking and contract negotiation, while roles, responsibilities and accountability mechanisms are made explicit. The second scheme concerns the contracting by local governments of DHMT to provide support services, supervision and regulatory tasks; part of the contract is for technical assistance by DHMT to local governments in the area of human resources. This scheme is being piloted and not yet evaluated. The third instrument is part of the national development investment fund that exists since 2006 and provides incentives to local governments to improve service delivery. Local governments can access additional funds based on actual improvements in key health indicators.

Through the three schemes, it is expected that health worker motivation and performance will improve, along with improvements in overall governance and accountability relations within the decentralized system. And in cases where the legislation of devolution policies does not provide sufficient clarity on the division of responsibilities, the performance-based financing approach may formalize responsibilities and enhance horizontal and downward accountability. The actual functioning and effects of these schemes have not yet been assessed.

#### Politics and patronage

A final remark related to capacity is the issue of politics and elite capture. It should be noted that decentralization does not eliminate the politics of human resource management. It simply shifts politics from the national to the local level or from the Ministry of Health to local government. Local politicians and bureaucrats, like their national counterparts, face similar obstacles and may have only weak incentives to improve the functioning of the system [2]. In Mali, patronage in the selection process of health workers is common, whether the employer is a local politician, a bureaucrat or a community health association. This has also been seen in other countries, for example in China [4]. This means that the debate should go beyond the risks of devolution to include a broader view on governance in the health sector and more attention for the political economy of HRH.

Similarly, decentralization design should also take into account political incentives and the potential effect of patronage. Partial devolution can be counterproductive, as observed by Khemani [7] in the case of Nigeria. Limited discretion (e.g. over staff recruitment, hiring, firing) of the local institution generates demotivation, lack of ownership and encourages corruption among local authorities [7]. Continuous interference by central government in local HRH issues in Tanzania reduced the autonomy of local authorities, which also reduced the effectiveness of decentralized management [3]. Although this has not yet been studied in Mali, the partial transfer of responsibilities under devolution could have a similar negative impact on the commitment of local authorities.

## Conclusion

Decentralization reforms are complex and dynamic processes and the outcomes for improved HRH are not yet fully known, in particular in the context of francophone West African countries that only recently established locally elected authorities. But also in more advanced decentralized systems, research on the effects or potential of devolution on human resources for health at the local level is limited. Measuring impact is further complicated by the co-existence of different forms of decentralization and the introduction of related health reforms.

The conclusions we can draw from this discussion is that the question is not whether to decentralize or not. The key issue to address is local accountability mechanisms. The redistribution of resources across actors and government levels will not solve weak public accountability of decisions made that affect HRH. Political economy needs to be taken into account and additional measures need to be put in place to provide incentives for local governments, providers and regulators to make the system work. A first step would be to harmonize decentralization and health policies and clarify authority and accountability relations.

Steps taken to support devolution in Mali, particularly the introduction of the local government service for health staff, the scaling up of a comprehensive capacity development programme and the introduction of performance-based financing, confirm the commitment of the Malian government to strengthening the local management of human resources. A preliminary assessment at the operational level suggests that devolution offers considerable opportunities for improving the responsiveness of health services, staff recruitment and retention and downward accountability. However, the partial transfer of responsibilities to the local level, which results in unbalanced accountability relations, seems to undermine the opportunities created at the local level.

This article aims to contribute to the current debate in Mali. However, providing concrete recommendations for the way forward is impossible without a more systematic analysis of policies and their implementation. The effects of decentralization on HRH at the local level need to be closely monitored in order to collect more evidence on what works and what does not.

## Competing interests

The authors declare that they have no competing interests.

## Authors' contributions

EL and DD were involved in the preparation of this article, with EL focusing on the literature review and outlining of the content and DD focusing on collecting policy documents and examples from Mali. A joint analysis was done with a few interviews with key stakeholder in Mali by both EL and DD. The manuscript was drafted by EL and reviewed and complemented by DD. Both authors read and approved the final manuscript.
